# Are the problem spaces of economic actors increasingly virtual? What geo-located web activity might tell us about economic dynamism

**DOI:** 10.1371/journal.pone.0239256

**Published:** 2020-09-17

**Authors:** Timothy R. Wojan, Timothy F. Slaper

**Affiliations:** 1 Resource and Rural Economics Division, Economic Research Service, Washington, DC, United States of America; 2 Indiana Business Research Center, Indiana University, Bloomington, Indiana, United States of America; TED University, TURKEY

## Abstract

The principal questions this research will address are: 1) whether a higher propensity to visit websites of interest to actual or nascent entrepreneurs is associated with higher rates of new firms births in an area; 2) whether a higher propensity to visit websites of interest to those working on design problems is associated with the quality of business in terms of orientations toward design or innovation; and 3) whether a higher propensity to visit websites of interest to those pursuing arts as an avocation is associated with an increased ability to find nonobvious solutions that might be manifest in business quality. The unique data that allow examining these questions were compiled from billions of web hits by geo-located devices. These data are combined with both detailed establishment level data with reliable information on the innovation and design orientation of firms, and a longitudinal census of all establishments with a formal credit relationship in the U.S. The findings confirm that businesses located in areas with a high propensity to visit design and arts avocation websites are more likely to pursue more far ranging innovation and are more likely to integrate design into their innovation processes. Firm birth rates are higher in areas with a high propensity to visit websites of interest to entrepreneurs, and the existence of high growth firms is strongly associated with demonstrated interest in design and arts avocation websites. The possible uses of these nontraditional measures as indicators of economic dynamism are discussed.

## Introduction

The objective of this research is to investigate whether interests expressed through web behavior provide new insight regarding the location of economic dynamism expressed as faster firm formation or higher firm innovation rates. If these unconventional data regarding virtual experience are associated with economic dynamism, then new opportunities to nowcast economic phenomenon in real time or to inform local economic development dashboards emerge [1, 2].

The economic geography of innovation has traditionally been concerned with those place attributes, and the types of non-virtual interaction across space, that explain higher rates of innovation. The idea that some places are better at facilitating or promoting innovation was first rigorously studied by Alfred Marshall in 1890 to explain the economic dynamism of the British industrial districts. Nearly 130 years later the idea that “the secrets of industry are in the air” is still a central tenet that guides research questions and government programs focused on regional innovation, premised on constructs such as localization, agglomeration, and information spillovers [3, 4]. The clustering of industry in a particular place is thought to engender a rich exchange of knowledge—generated from both success and failure—allowing participants in that cluster to learn and innovate more quickly than peers with few or no competitors cum collaborators. In the 1890s, the physical space containing the industrial district also largely defined the problem space for cluster participants, as physical proximity was the principal vector for the transmission of information. In 2020, the assumption that the physical space largely defines the problem space is surprisingly resolute given the enormous reduction in the cost of communications accompanied by an enormous growth in the capability for rich transmissions [5]. However, the one thing that guarantees the centrality of physical proximity no matter how the processes of innovation may have changed is the wealth of data available to study it.

In contrast, data to examine how information and knowledge from non-local sources might affect economic dynamism are quite rare. Primary data collection to study where businesses get their information finds that local sources are not always the most valuable [6], consistent with Marshall’s conjecture that information from global production would displace information from local production as the cost of communication declines [5].

This research uses nontraditional, non-intrusive data collection techniques to examine whether information gleaned from the Internet on general problems related to entrepreneurship and design are in fact associated with faster rates of new firm formation and business orientations toward design and innovation. Evidence that areas actively tapping these information sources do demonstrate greater economic dynamism would support Marshall’s conjecture regarding the eventual death of distance that might be summarized as “some secrets of industry are now also in the ether.” Failure to identify an association between web activity related to entrepreneurship and design, and economic dynamism, would lend more credence to the claim that “the secrets of industry that matter most are still in the air.”

Alternatively, a propensity to visit websites focused on entrepreneurship or design may merely provide an indicator of intensity of interest in these subjects. This interest might be associated with a greater capability for accessing and absorbing non-virtual information through traditional vectors such as proximity. That is, evidence of an association between web activity and economic dynamism might still support the claim that “the secrets of industry that matter most are still in the air” if web activity serves mainly as an indicator of intensity of interest. The analysis is agnostic as to whether the dominant mechanism provided by web behavior is the transfer of information or identification of capability/interest. The main interest is assessing web behavior provides a reliable indicator of economic dynamism.

The unique data that allow answering the research question are provided by DStillery, a marketing firm that uses advanced data science techniques to identify customers or “crafted audiences” for a diverse set of clients based on web behavior distilled from billions of website visits each day. The geocoding of devices allows examining how the presence of these “crafted audiences” differ across space. The Indiana University Regional Economic Development (IU RED) project provided DStillery with a set of websites demonstrated to be popular and believed to be valued sources of information for people interested in starting, managing, or growing a business. The compiled hits to these websites from geolocated devices are then modeled to produce an index by ZIP code that indicates the relative propensity of area residents to click on those websites. This represents the “E-ship Audience.”

In addition, a list of websites demonstrated to be popular and believed to be valued sources of information on design—whether web design, graphic design, or product design—was also provided to build a “Design Audience.” Solving design problems is a fundamental input to comparative advantage in the innovation economy. Research by the OECD [7] and Wojan and Nichols [8] demonstrate that businesses with a high commitment to design demonstrate superior innovation performance. A third audience is constructed to test the hypothesis—with strong anecdotal support—that exercising the artistic imagination outside one’s core discipline increases the chances of arriving at nonobvious, truly innovative solutions within that discipline [9, 10]. A list of websites believed to be valued sources of information for those pursuing a wide range of arts as an avocation was used to construct an “Arts Avocation Audience.”

We combine the DStillery crafted audiences with the 2014 Rural Establishment Innovation Survey (REIS), collected by the U.S. Department of Agriculture’s Economic Research Service (ERS). The REIS is a large nationally representative sample of businesses in tradable sectors with at least 5 employees and is well-suited for examining how the interests expressed by these crafted audiences is associated with the innovation and design orientation of local businesses. The unique contributions of the REIS are the reliable measures of self-reported substantive innovation and design-integrated activities that have been shown to be associated with superior business performance [8]. Although the crafted audience indexes do not provide explicit information on the interests of employees in an individual business, they do provide proxies for the entrepreneurial, design, and artistic milieu of the local area. Strong associations between business strategy and these local milieux, after controlling for relevant local characteristics, would provide evidence of the value of non-traditional data for understanding economic phenomena.

An out-of-sample test of the value of these data is performed using the National Establishment Time Series (NETS) data, a longitudinal census of all businesses in the U.S. with a formal credit relationship. Critical threshold values for the crafted audience indexes identified in REIS are used to classify the much larger number of ZIP codes in NETS into quantiles. If performance measured in terms of firm births, employment growth, or number of high growth firms is consistently associated with higher levels of the various indexes, then it is plausible to conclude that the indexes provide useful information on where the innovation and design orientations identified using the REIS data are likely to be most prevalent. In this way, the insights from a limited, representative sample of establishments might be extended to localities throughout the nation.

The potential productiveness of these crafted audiences for providing near real-time metrics on aspirations and motivations for entrepreneurial, design, and innovation pursuits concludes the discussion. Including this information in a dashboard for local leaders containing information on development opportunities and challenges is the objective of the IU RED project. The most effective strategies for pursuing economic development are likely to differ based on local proclivities for entrepreneurship and the fervor of local design and artistic imaginations.

## Constructing the crafted audiences

DStillery is a predictive marketing intelligence firm that anonymously collects, classifies, and disseminates behavioral data. While DStillery is well established in the digital marketing ecosystem, the application of their data linking individual website behavior to economic activity is novel. DStillery's digital data is collected as a proxy for real-world behaviors. Marketers use these data to optimize which devices see ads at times relevant to a consumer [11].

DStillery can create an analytical category based on the websites a marketer or researcher may think pertains to a particular constituency. DStillery captures a representative sample of devices that visit these websites and finds the highest scoring features to create a model that scores devices based on their affinity to the behavior of interest [12], in this case the E-ship, Design or Arts Avocation audiences. The predictive models use logistic regression trained with stochastic gradient descent [13]. The score of that set of devices is compared to a random sample of devices across the internet. Device scores are aggregated at the ZIP code level, based on the predicted "home" location of each device according to a probabilistic model that takes into account time of day and frequency of visit to a discrete location.

The predicted home location (all desktop, laptop, mobile and tablet devices used by an individual or household) is critical to accurately estimating geo-located affinities. DStillery uses a device graph that probabilistically connects a user’s devices to one another using IP addresses, location data, and time as inputs. Devices that are seen on the same wifi IP addresses within a specific period of time are connected. However, if numerous devices are seen on one IP address, likely representing a public access node such as a coffee shop or library, the IP address is not used to expand the device graph. Other data quality filters detect anomalous and likely fraudulent web impressions. If a device impression occurs in one locale followed minutes later by an impression 1,000 miles away the device is filtered out. Similarly, bot nets that generate fraudulent ad impressions are identified through a machine-learning process that identifies clusters of fraudulent sites and devices that visit them. These devices are also filtered out of the dataset [14].

The E-ship audience was created using a list of 100 relevant websites from the Kauffman Foundation website, plus several entrepreneurial website guides provided by the Indiana Business Research Center, which DStillery used to observe and measure traffic (see S1 Appendix). These data for E-ship affinity were collected at the ZIP code level.

The Design and Arts Avocation audiences were creating using lists generated by ERS researchers with input from the National Endowment for the Arts (see S2 Appendix). The definition of “design” and “art” were very broad for this first cut with design including graphic, fashion, website, and industrial design sites, and art including fine, performing, and applied art ranging from oil painting, poetry, and ballet to home foundries, wood working, and blues guitar. Traffic to these sites was also observed and measured at the ZIP code level.

Despite the novelty of these data, most observers of the innovation economy have strong priors on where they would expect to see the highest scores for the E-ship index. Clearly, if entrepreneurial hotspots such as the Bay Area, Austin, or Seattle generated low scores on the E-ship index one would be left to wonder precisely what the index is measuring. Priors with respect to design and arts avocations are much less clear as journalism on arts and design pales in comparison to reports on fast growing business start-ups, and the arts avocation index is specifically targeted to amateur pursuits. Followers of NEA’s periodic Survey of Public Participation in the Arts would expect to see Colorado have a disproportionate share of hotspots given that more than 60% of adult residents of Colorado consistently claim to personally perform or create artworks, the highest of any state. Northern New England is another anticipated hotspot based on NEA data.

### Mapping where web interest in entrepreneurship, design, and arts avocations are the highest

**Fig 1:**
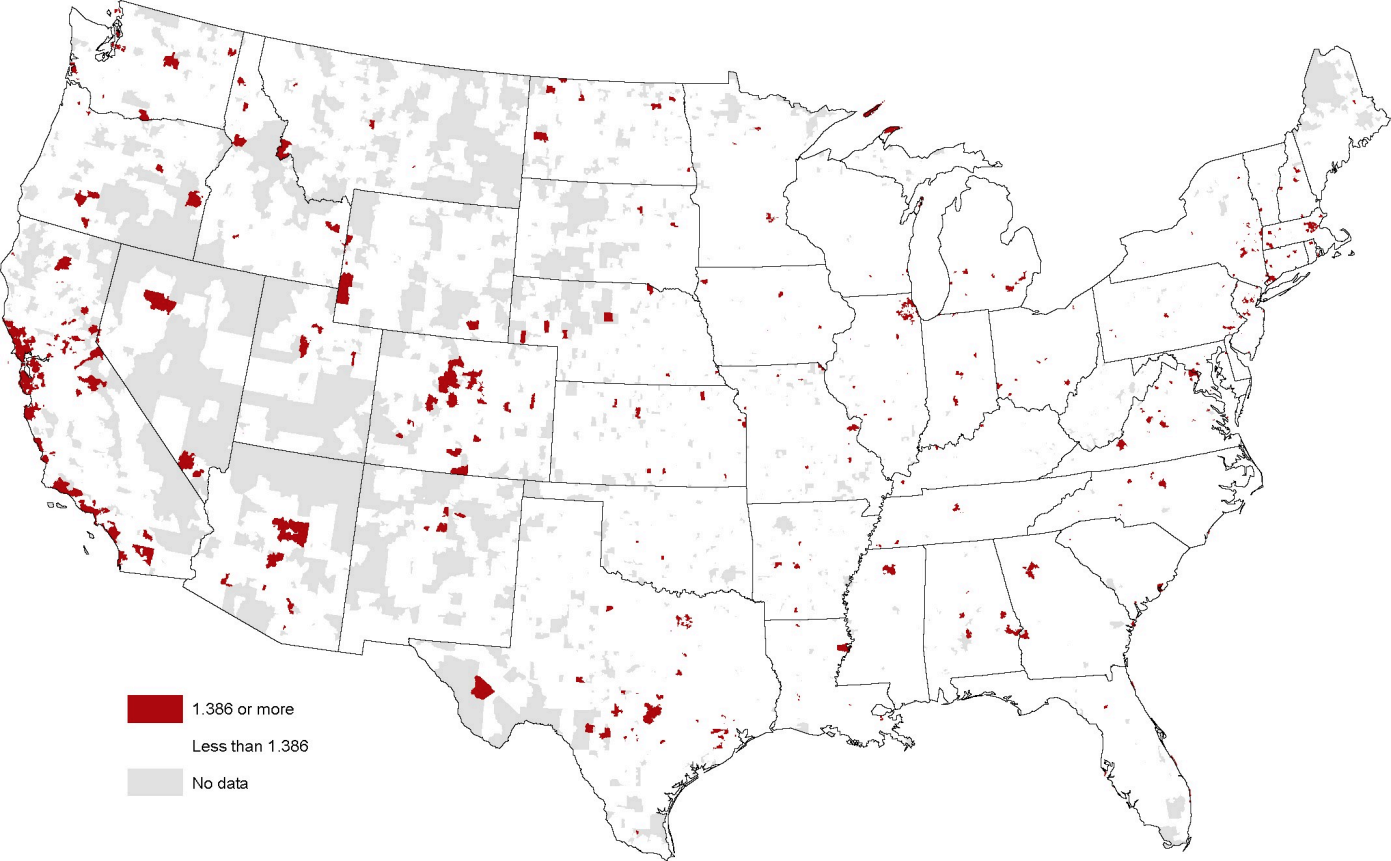
Entrepreneurship hotspots (index > 1.386) mapped at ZIP code level, January 2018. Sources: Esri, DStillery Custom Crafted Audience. Maps were created using ArcGIS® software by Esri. ArcGIS® and ArcMap™ are the intellectual property of Esri and are used herein under license. Copyright Esri. All rights reserved. For more information about Esri® software, please visit www.esri.com.

Do the custom crafted audience indexes comport with these priors? Mapping the index at ZIP Code level can create the false impression that sparsely populated areas are more likely to be hotspots given the larger area they command on the map. But even acknowledging this potential visual bias, the map does a good job of confirming our priors. Entrepreneurial hotspots (comprising zip codes in the top 5% of the E-ship Index) in the Bay Area, Austin, and Seattle are accompanied by hotspots near Boston, Research Triangle, DC, Atlanta, Nashville, Chicago, Denver, Salt Lake City, Los Angeles and San Diego (see Fig 1). The index can be interpreted as an odds ratio where residents in hotpots would be at least 38.6% more likely to visit an entrepreneurship website. Interestingly, the protocol also picks up clusters of entrepreneurial hotspots that are not the usual suspects. Amenity migrants would appear to provide an ample supply of lone eagle entrepreneurs in mountain areas west of Denver. University towns in Indiana (Bloomington) and Wisconsin (Madison) suggest a different story explaining entrepreneurial fervor. However, the presence of these hotspots in every state in the Union—but often not in a capital or large city—suggests a multitude of different possible stories. Whether the index is strongly associated with more robust start-up activity will be examined below.

**Fig 2:**
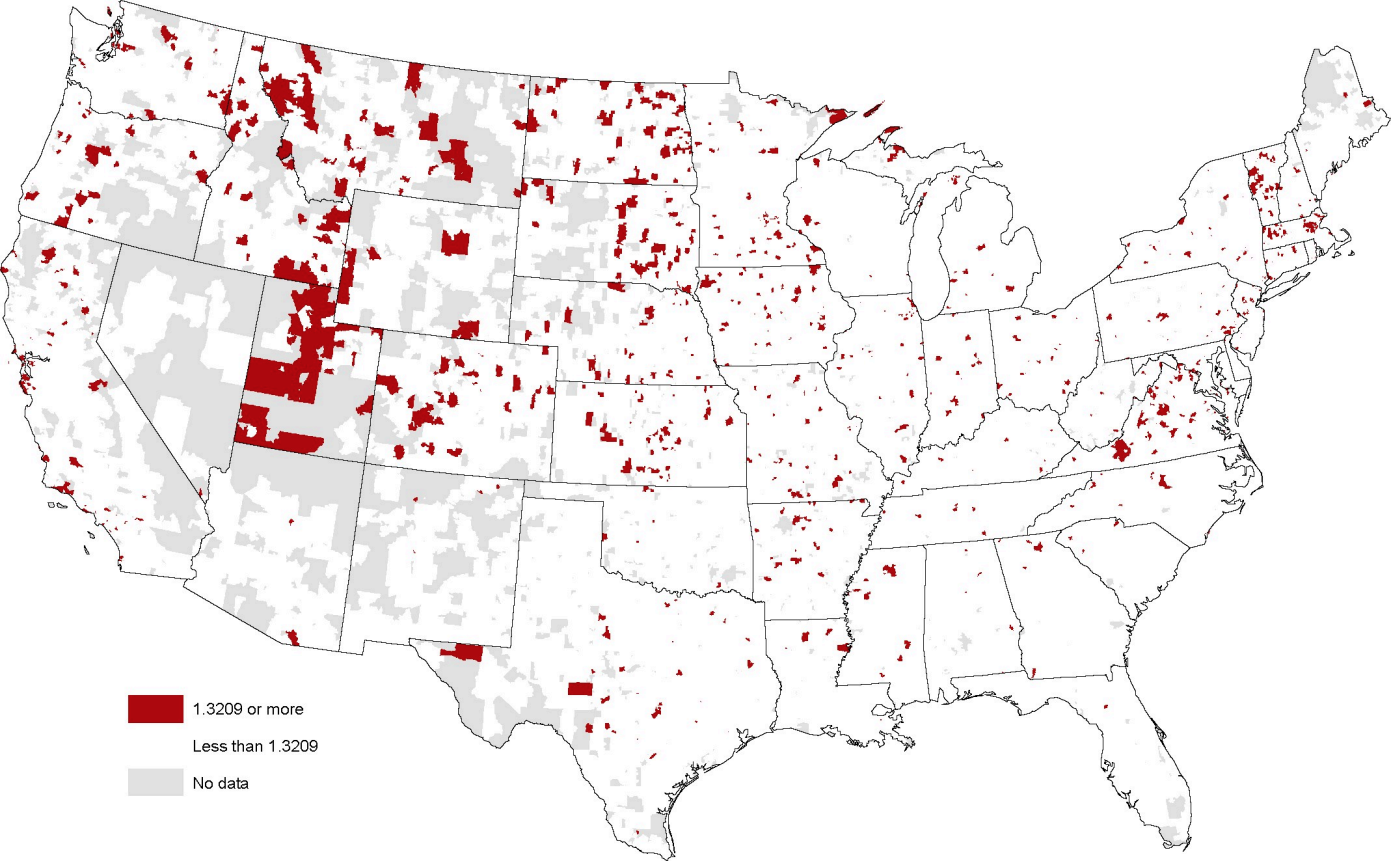
Arts avocation hotspots (index > 1. 32) mapped at ZIP code level, January 2018. Sources: Esri, DStillery Custom Crafted Audience. Maps were created using ArcGIS® software by Esri. ArcGIS® and ArcMap™ are the intellectual property of Esri and are used herein under license. Copyright Esri. All rights reserved. For more information about Esri® software, please visit www.esri.com.

Arts Avocation hotspots are mapped in Fig 2. The same top 5% threshold is used so the larger covered area on the map relative to the E-ship Index suggests the hotspots tend to locate in less densely populated zip codes. Priors regarding numerous hotspots in Colorado and Vermont are confirmed but the standout region is Utah that has the largest collection of contiguous hotspots of anywhere in the Nation. The relative absence of Arts Avocation hotspot in large cities like New York or LA recasts Richard Florida’s creative class as arts consumers and spectators rather than hobbyist creators and performers [15]. The absence of clear large city hotspots gives the Arts Avocation index a footloose feel, particularly with hotspots in remote, sparsely populated areas in the Dakotas, Nebraska, and Kansas. The fact that an association with economic dynamism is not readily apparent from the map increases curiosity over what the statistical analysis may reveal.

**Fig 3:**
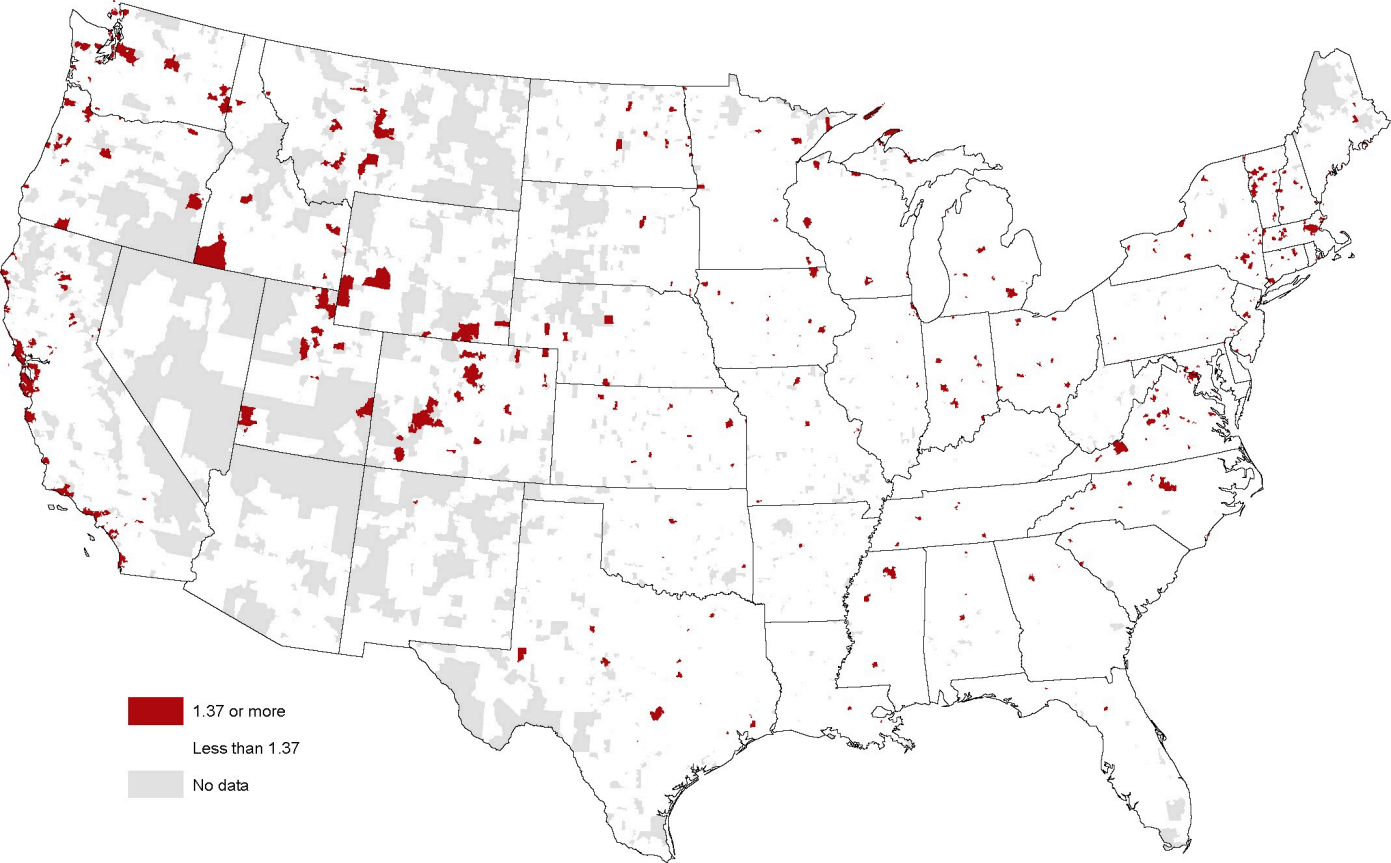
Design hotspots (index > 1.37) mapped at ZIP code level, January 2018. Sources: Esri, DStillery Custom Crafted Audience. Maps were created using ArcGIS® software by Esri. ArcGIS® and ArcMap™ are the intellectual property of Esri and are used herein under license. Copyright Esri. All rights reserved. For more information about Esri® software, please visit www.esri.com.

Priors on the Design hotspots are aided by the assumed stronger vocational focus than either the E-ship index (e.g., many aspirational entrepreneurs visiting these sites may never start a business) or the Arts Avocation index. It is reasonable to assume that places with a reputation for design-oriented business, or as the center for design consultancy, should host hotspots. The San Francisco Bay Area (home of the IDEO design consultancy) is clearly represented in the Fig 3 map. And there are hotspots that line up with E-ship hotspots in other big cities such as LA, Seattle, Austin, Chicago, and Boston. The main difference with the E-ship index map is that the urban Design hotspots tend to be contained within a smaller number of ZIP Codes within an MSA. However, as Wojan and Nichols [8] found, design is not solely an urban phenomenon. Montana, Colorado, and Vermont all have a number of design hotspots in rural areas of these states.

The maps are compelling in demonstrating the dispersion of places with high interest across the nation. While it is reassuring that the method picks up some known entrepreneurial and innovation hotspots such as Austin and the San Francisco Bay Area, the mappings are much more complex than mere clustering around global cities. One characteristic the maps hint at are possible strong correlations between the indexes. A correlation matrix (Table 1) confirms this is in fact the case. On the positive side this may indicate that the indexes are all picking up a common latent factor such as a search for novelty or openness to new ideas that characterize these places. On the negative side, this may make it more difficult to isolate the independent effects of an interest in entrepreneurship with new firm starts and an interest in design/arts with the innovation orientation of local businesses. A third possibility—that the level of the indexes is merely an artifact of broadband/4G saturation in some places and limited service in others—is effectively dismissed by high levels in unexpected places such as North Dakota. The possible association between the indexes and business strategy is an empirical question that is examined next.

**Table 1:** Correlation of crafted audience indexes across U.S. ZIP code geography.

Pearson Correlation Coefficients			
Prob > |r| under H0: Rho = 0			
Number of Observations			
	E—ship_index	Design_index	Arts_Avocation_index
**E—ship_index**	N = 27557		
**Design_index**	0.73119	N = 27727	
	< .0001		
	27432		
**Arts_Avocation_index**	0.55236	0.62524	N = 27802
	< .0001	< .0001	
	27504	27601	

Source: Dstillery Custom Crafted Audience.

## The association between crafted audience web behavior and the innovation/design orientation of local businesses

Despite the suggestive maps and the strong correlation across the indexes, it is important to keep the different geographical scales of the indexes and establishment level data to which they will be applied in perspective. The possible behavioral or strategic manifestations associated with the ZIP code level indexes will be assessed at the establishment level, based on responses from businesses in nonfarm tradable sectors with 5 or more employees in the 2014 ERS Rural Establishment Innovation Survey. The ZIP code level index may or may not provide an accurate representation of the interests of employees within the business. The research question is whether interests measured at the area level are associated with design or innovation orientations at the business level.

The other challenge presented by merging the DStillery indexes with REIS is the apparent temporal inconsistency: the indexes constructed on the basis of web traffic in January 2018 are being used to predict (or least examine associations with) behavior in 2014. Reverse causality arguments—i.e., that the establishment behavior of interest in 2014 is influencing the 2018 index—need to be kept in mind in assessing the findings.

The main purpose of REIS was to assess whether rural businesses were able to innovate at the same rate as their urban peers. This objective required a reliable normative measure of innovation, something that other self-reported innovation surveys such as the EU Community Innovation Survey (CIS) or the U.S. Business R&D and Innovation Survey (BRDIS) are not concerned with. REIS supplemented the self-reported innovation questions found in CIS and BRDIS with two sets of auxiliary questions; where affirmative responses were believed to be associated with incremental innovation and substantive (or more far-ranging) innovation, respectively. Latent class analysis (LCA) was then used to determine membership in a Non-Innovator, Incremental Innovator, and Substantive Innovator class probabilistically [16]. Although estimating the design orientation of establishments was not an objective of the survey at the outset, an adequate number of questions on design in the survey did allow developing a *post hoc* latent class analysis to determine membership in rungs of a design ladder developed by Statistics Denmark, corresponding to respondents with No Systematic Approach to Design, respondents using Design as a Last Finish Before Going to Market, or respondents Integrating Design Throughout the Development Process [8]. Details of the protocol and validation tests for both the innovation LCA and design LCA are available in the supplement to Wojan and Nichols [8].

Both the innovation and design orientations were found to be right tail phenomena with 20% (rural) to 30% (urban) of establishments classified as substantive innovators and 5% (rural) to 10% (urban) of establishments classified as design integrated [8, 16]. More than 80% of the design integrated establishments were also classified as substantive innovators. Establishment-level estimates of the impact of design or innovation orientation on growth are not yet available but self-reported performance of the three years preceding the survey support the presumption that these are high performance businesses. Design integrated establishments were more than twice as likely to enter export markets than their design last finish peers and 10 times more likely than their no systematic design peers. Design integrated establishments also reported significantly higher likelihood of expanding market share. Making up a much larger share of the population of establishments, the performance advantages of substantive innovators were not as dramatic relative to nominal and non-innovators but were still substantial Substantive innovators were more than twice as likely to enter export markets than nominal innovators and 3 times more likely than non-innovators. And while three-quarters of substantive innovators reported increasing market share, only a half of nominal innovators, and 40% of non-innovators reported the same.

The research question is parallel to the one posed in Wojan and Nichols [8]: Are local characteristics associated with the probability that a business incorporates design in its business strategy? The principal variable of interest in Wojan and Nichols [8] was the presence of performing arts organizations, limited to nonmetro counties. The analysis also controlled for educational attainment, the presence of a 4-year college, housing cost, natural amenities, and population density. In the current analysis we merely substitute the crafted audience indexes for the presence of performing arts organizations. The correlation between the crafted audience index and the presence of a performing arts organization in nonmetro counties was 0.168 with a p-value of <0.0001. The added value of the crafted audience index is that it allows extending the analysis to metropolitan counties as the substituted variable has the variation required for estimation that was not available with the near ubiquity of metro performing arts organizations.

The incorporation of design in a business is modeled as an ordered logistic regression as establishments classified as having no systematic approach to design have a much lower probability of being classified as design integrated relative to design last finish establishments. The latent class structure estimated is consistent with a clear gradation of design intensity across the 3 classes. Specification testing revealed that all three indexes were positively and significantly associated with a higher probability of incorporating design, at least for some of the subsamples defined by metro/nonmetro status and entrepreneurial orientation. Alternatives to including a single index were investigated, including a principal component derived from the three indexes and multiplicative and additive combinations of the indexes. The option that produced the most consistent results across the subsamples was derived by multiplying the Design Index by the Arts Avocation Index (ArtsDesignIndex). Results from this estimation, for all establishments in the sample, are provided in Table 2.

**Table 2:** Ordered logistic regression of design orientation for all establishments in REIS.

	Full Sample	Odds Ratio
Intercept DesInt	-6.4370	
	(< .0001)	
Intercept DesFin	-4.0492)	
	(0.0002)	
EstablishmentSize	0.0149	1.015
	(< .0001)	
LogPopDensity	0.1874	1.206
	(< .0006)	
4YrCollege	-0.0125	
	(0.9453)	
PctCollGrad25_44	0.9359	
	(0.3176)	
MdContractRent	-0.00043	
	(0.3167)	
NatAmenityScale	0.0374	
	(0.1098)	
ArtsDesignIndex	1.0861	2.963
	(< .0008)	
Metro County	-0.2669	
	(0.2139)	
Entrepreneurial Firm	0.6792	1.972
	(< .0001)	
N	6712	
Percent Concordant	70.2	
Percent Discordant	29.4	
Percent Tied	0.4	

Source: 2014 ERS Rural Establishment Innovation Survey, DStillery Custom Crafted Audience, Census ACS.

Entrepreneurial establishments were identified by asking if the entity was started to introduce a new product or service to the market. Nonentrepreneurial establishments would include branches of multi-unit plants, franchises, or filling demand for a standard product or service.

Estimation using SAS Proc Surveylogistic with robust clustered standard errors at county level.

P-values are in parentheses.

3-digit NAICS industry controls not shown.

The ArtsDesignIndex is associated with a higher probability of being classified as Design Integrated relative to Design Last Finish, or Design Last Finish relative to No System Design. The magnitude is substantial with the odds ratio suggesting that a one unit increase in the ArtsDesignIndex is associated with an establishment being close to 3 times more likely to be classified in a higher design oriented category. Entrepreneurial establishments are twice as likely to be classified as more design oriented relative to nonentrepreneurial establishments. The earlier analysis [8] used the presence of performing arts organizations as an indicator variable of local artistic milieu. The presence of performing arts organizations increasing the likelihood of higher design orientation by 50% for entrepreneurial rural establishments. The two advantages the ArtsDesignIndex has over the performing arts organization indicator variable is the ability to estimate an effect for urban areas and a much closer conceptual connection to the demonstrated interest of local residents in the arts. The estimated effect is both more general and arguably of larger magnitude.

Establishment size and population density are the two other variables with statistically significant coefficient estimates. Smaller establishments may have fewer resources for adopting a systematic approach to design or, for truly design intensive small firms, design capabilities may be more idiosyncratically located within a business founder or owner. The significant population density estimate suggests that design orientation is positively associated with agglomeration or localization. Population density and the metro indicator variable are strongly correlated (Pearson correlation coefficient of 0.599). While “metro” may be more evocative of a city, this category can include suburban and ex-urban bedroom communities and counties covering large land areas but with settlements with more than 50,000 residents. Population density provides a much tighter measure of urbanization. Given the rural focus of the earlier analysis [8], it is instructive to examine the association between design orientation and the ArtDesignIndex within rural (nonmetropolitan) and urban (metropolitan) domains.

The results in Table 3 confirm that establishment size is consistently associated with design orientation within all domains. None of the other independent variable coefficient estimates are statistically significant in all four equations. Most notably, within domain estimates for the ArtsDesignIndex variable are not significant for either rural domain but are significant for both urban domains. The large magnitude of the coefficient estimate in the Urban Nonentrepreneurial domain is considerably larger than the estimate in the aggregate equation. The estimated coefficient for the human capital variable (PctCollGrad25_44) is large for the entrepreneurial establishment domain in both rural and urban areas, an association masked in the aggregate equation.

**Table 3:** Ordered logistic regression of design orientation for domains defined by entrepreneurial/non-entrepreneurial and rural/urban status.

	Rural Entrep. Est	Rural Entrep. Odds	Rural NEntrep. Est	Rural NEntrep. Odds	Urban Entrep. Est	Urban Entrep. Odds	Urban Nentrep Est	Urban Nentrep Odds
Int. DesignIntegrated	-18.4909		-5.6972		-20.111		-5.9794	
	(< .0001)		(< .0001)		(< .0001)		(< .0001)	
Int. DesignLastFinish	-15.58		-2.7143		-17.738		-3.4127	
	(< .0001)		(0.0004)		(< .0001)		(0.0078)	
EstablishmentSize	0.0152	1.015	0.0163	1.016	0.0137	1.014	0.0175	1.018
	(< .0001)		(< .0001)		(0.0003)		(< .0001)	
LogPopDensity	0.0717		-0.00046		0.145		0.2724	1.313
	(0.2725)		(0.9945)		(0.1149)		(0.0055)	
4YrCollegePresent	0.0184		0.1351		0.2163		-0.3484	
	(0.8912)		(0.3361)		(0.4055)		(0.2486)	
PctCollGrad25_44	1.8258	6.208	-0.3		3.156	23.476	-0.9515	
	(0.0418)		(0.7589)		(0.0133)		(0.5797)	
MedianContractRent	0.00148	1.001	0.00164	1.002	-0.0003		-0.0004	
	(0.0156)		(0.0218)		(0.5900)		(0.5516)	
NaturalAmenityScale	0.0194		-0.0484		0.0503		0.0399	
	(0.4471)		(0.0762)		(0.1214)		(0.2873)	
ArtsDesignIndex	0.2507		0.2906		0.6914	1.997	1.7585	5.804
	(0.4781)		(0.3320)		(0.0991)		(0.0021)	
N in Domain	2359		3458		910		1106	
Percent Concordant	72.1		69.8		68.9		68.6	
Percent Discordant	27.5		29.5		30.8		30.9	
Percent Tied	0.4		0.7		0.4		0.5	

Source: 2014 ERS Rural Establishment Innovation Survey, DStillery Custom Crafted Audience, Census ACS.

Entrepreneurial establishments were identified by asking if the entity was started to introduce a new product or service to the market. Nonentrepreneurial establishments would include branches of multi-unit plants, franchises, or filling demand for a standard product or service.

SAS Proc Surveylogistic used to produce valid domain estimates of standard errors that are clustered at county level.

P-values are in parentheses.

3-digit NAICS industry controls not shown.

The orientation towards innovation in a business is also modeled using an ordered logistic regression with establishments classified as non-innovators, incremental or nominal innovators, and more far ranging or substantive innovators. Similar to the design ladder, the latent class structure estimated is consistent with a clear gradation of innovation intensity across the 3 classes. Specification testing revealed that all three indexes were positively and significantly associated with a higher probability of an incremental or substantive innovation orientation, at least for some of the subsamples defined by metro/nonmetro status and entrepreneurial orientation. As was true in the design ladder exercise, the option that produced the most consistent results across the subsamples was derived by multiplying the Design Index by the Arts Avocation Index (ArtsDesignIndex). Given the close association between the Design Integrated class and the Substantive Innovator class this result is not surprising. Results from this estimation for the entire sample are provided in Table 4.

**Table 4:** Ordered logistic regression of innovation orientation for all establishments in REIS.

	Full Sample	Odds Ratio
Intercept DesInt	-3.7851	
	(< .0001)	
Intercept DesFin	-2.0792	
	(0.0001)	
EstablishmentSize	0.0247	1.025
	(< .0001)	
LogPopDensity	-0.013	
	(0.7910)	
4YrCollege	-0.1663	
	(0.2618)	
PctCollGrad25_44	0.8838	
	(0.2785)	
MdContractRent	0.000437	
	(0.2480)	
NatAmenityScale	-0.0109	
	(0.6112)	
ArtsDesignIndex	0.7662	2.152
	(0.0105)	
Metro County	0.25	
	(0.1076)	
Entrepreneurial Firm	0.577	1.781
	(< .0001)	
N	6712	
Percent Concordant	65	
Percent Discordant	34.6	
Percent Tied	0.4	

Source: 2014 ERS Rural Establishment Innovation Survey, DStillery Custom Crafted Audience, Census ACS.

Entrepreneurial establishments were identified by asking if the entity was started to introduce a new product or service to the market. Nonentrepreneurial establishments would include branches of multi-unit plants, franchises, or filling demand for a standard product or service.

Estimation using SAS Proc Surveylogistic with robust clustered standard errors at county level.

P-values are in parentheses.

3-digit NAICS industry controls not shown.

Given that both Substantive Innovators and Incremental Innovators are more common than Design Integrated and Design Last Finish establishments, respectively, the ability of the area level ArtsDesignIndex variable to predict establishment level business strategy related to innovation with roughly similar levels of precision is surprising. However, the fact that the overwhelming majority of Substantive Innovators and the clear majority of Incremental Innovators have some commitment to design makes this finding credible [8]. The findings suggest that those areas where residents express little interest in design or arts avocation through their web behavior are less likely to spawn innovative businesses. Neither population density nor metro status are statistically significant suggesting that agglomeration or localization may be less important for ‘grassroots innovation’ than for design, at least as these constructs are defined in REIS.

Table 5 presents regression results for the four domains. Similar to the design regressions, the ArtsDesignIndex coefficient estimates are only significant in two of the four domains. Nonentrepreneurial establishments in both rural and urban areas were more likely to have a higher innovation orientation in those areas where residents are more likely to visit arts avocation and design websites. In the innovation regressions the human capital variable (PctCollGrad25_44) is only significant in the urban entrepreneurial domain. Innovation orientation is consistently associated with establishment size across all domains.

**Table 5:** Ordered logistic regression of innovation orientation for domains defined by entrepreneurial/non-entrepreneurial establishments and rural/urban status.

	Rural Entrep. Est	Rural Entrep. Odds	Rural NEntrep. Est	Rural NEntrep. Odds	Urban Entrep. Est	Urban Entrep. Odds	Urban Nentrep Est	Urban Nentrep Odds
Int. SubstantiveInnov	-4.5534		-4.8883		-2.8264		-3.5829	
	(< .0001)		(< .0001)		(0.0025)		(< .0001)	
Int. NominalInnov	-2.6982		-2.924		-1.0889		-1.8508	
	(0.0181)		(0.0002)		(0.2391)		(0.0182)	
EstablishmentSize	0.0189	1.019	0.023	1.023	0.0262	1.027	0.0269	1.027
	(< .0001)		(< .0001)		(< .0001)		(< .0001)	
LogPopDensity	0.1236	1.132	0.064		0.00181		-0.0696	
	(0.0210)		(0.2414)		(0.9827)		(0.3935)	
4YrCollegePresent	-0.0548		0.057		-0.131		-0.3463	
	(0.6541)		(0.6590)		(0.6188)		(0.1810)	
PctCollGrad25_44	-0.35		-0.3091		3.1658	23.708	-0.0991	
	(0.6733)		(0.7278)		(0.0156)		(0.9387)	
MedianContractRent	0.00112	1.001	0.00104	1.001	-5E-05		0.00099	
	(0.0192)		(0.0493)		(0.9320)		(0.1036)	
NaturalAmenityScale	-0.0104		-0.0434	0.958	0.00364		-0.0203	
	(0.6595)		(0.0471)		(0.8995)		(0.5974)	
ArtsDesignIndex	0.2129		0.6186	1.856	0.2532		1.0607	2.889
	(0.5062)		(0.0386)		(0.5702)		(0.0271)	
N in Domain	2359		3458		910		1106	
Percent Concordant	66.9		65.8		66.9		66.6	
Percent Discordant	32.7		33.7		32.7		33	
Percent Tied	0.4		0.6		0.4		0.4	

Source: 2014 ERS Rural Establishment Innovation Survey, DStillery Custom Crafted Audience, Census ACS.

Entrepreneurial establishments were identified by asking if the entity was started to introduce a new product or service to the market. Nonentrepreneurial establishments would include branches of multi-unit plants, franchises, or filling demand for a standard product or service.

SAS Proc Surveylogistic used to produce valid domain estimates of standard errors that are clustered at county level.

P-values are in parentheses.

3-digit NAICS industry controls not shown.

Comparing Tables 2 to 4 reinforces the commonality between design and innovation regarding the influence of the ArtsDesignIndex variable. Establishment size is also consistently associated with a higher design or innovation orientation. Differences between design and innovation are apparent in the importance of population density to these phenomena.

The methods used to arrive at a seemingly robust association between the ArtsDesignIndex variable and commitments to design and innovation does warrant skepticism regarding the generality of this result. Because the final estimation was arrived at after a number of alternative specifications of the crafted audience indexes, the nominal p-values are much lower than the real p-values. That is, as the number of tests increase the probability that the finding is unique to a particular sample also increases. Validating the results out of sample provides a hard test of the generality of the findings.

## Extending the micro-level analysis to the entire economy using NETS

The strategy used to test the generality of the logistic regression results to the whole economy is to define relevant thresholds for values of the ArtsDesignIndex given the detailed data in REIS, apply these thresholds to all ZIP codes in the US for which we have an ArtsDesignIndex, and then examine the relative performance of businesses in these ZIP codes given different levels of the index using the longitudinal census of businesses provided by the National Establishment Time Series (NETS). In addition to providing an external test of the regression results, this strategy also demonstrates a method for providing potentially relevant information for local areas that is not possible from micro-level datasets given the small number of observations for each area.

The method relies on two assumptions. First, as right tail phenomena, the relevant criteria for arriving at thresholds for the ArtsDesignIndex is differentiation across bins for an upper quantile. Given the rarity of design-integrated establishments we use observations above the 90^th^ percentile with respect to the probability of being so classified. For innovators we use observations above the 75^th^ percentile of being classified as a substantive innovator. Second, there is some equal quantile division using the ArtsDesignIndex that produces large differences between the top bin and all lower bins.

For each observation in REIS the latent class analysis estimates the probability that the establishment is a member of the Design-Integrated or the Substantive Innovator class. We use a 60% probability (better than even odds) of being classified as Design Integrated/Substantive Innovator as the relevant criterion for assessing membership at the 75^th^ (Innovation) and 90^th^ (Design) percentile. As a concrete example, if the REIS observations are partitioned into bins based on their ArtsDesignIndex value then a crisp differentiation of design integrated businesses would see all observations above the 90^th^ percentile meeting the 60% threshold in the top bin (businesses in ZIP codes with the highest level of the ArtsDesignIndex), and none of the observations above the 90^th^ percentile meeting the 60% threshold in the bottom bin (businesses in ZIP codes with the lowest level of the ArtsDesignindex). In contrast, a poor differentiation would see only small differences in the share of the top 10 percent meeting the 60% threshold. Such a result would raise doubts about the generality of the ArtsDesignIndex explaining differences in design orientation of businesses across ZIP codes.

The SAS command ‘Proc Rank’ was used to partition the REIS observations into 3 to 6 bins based on the value of the ArtsDesginIndex. ‘Proc Univariate’ was then used to assess differences at the 75^th^, 90^th^, 95^th^, and 99^th^ percentile observation in the probability of being classified as Design Integrated or a Substantive Innovator. The 3-bin partition produced the cleanest differentiation for the innovation probability, and the 4 bin partition produced the cleanest differentiation for the design probability.

**Fig 4:**
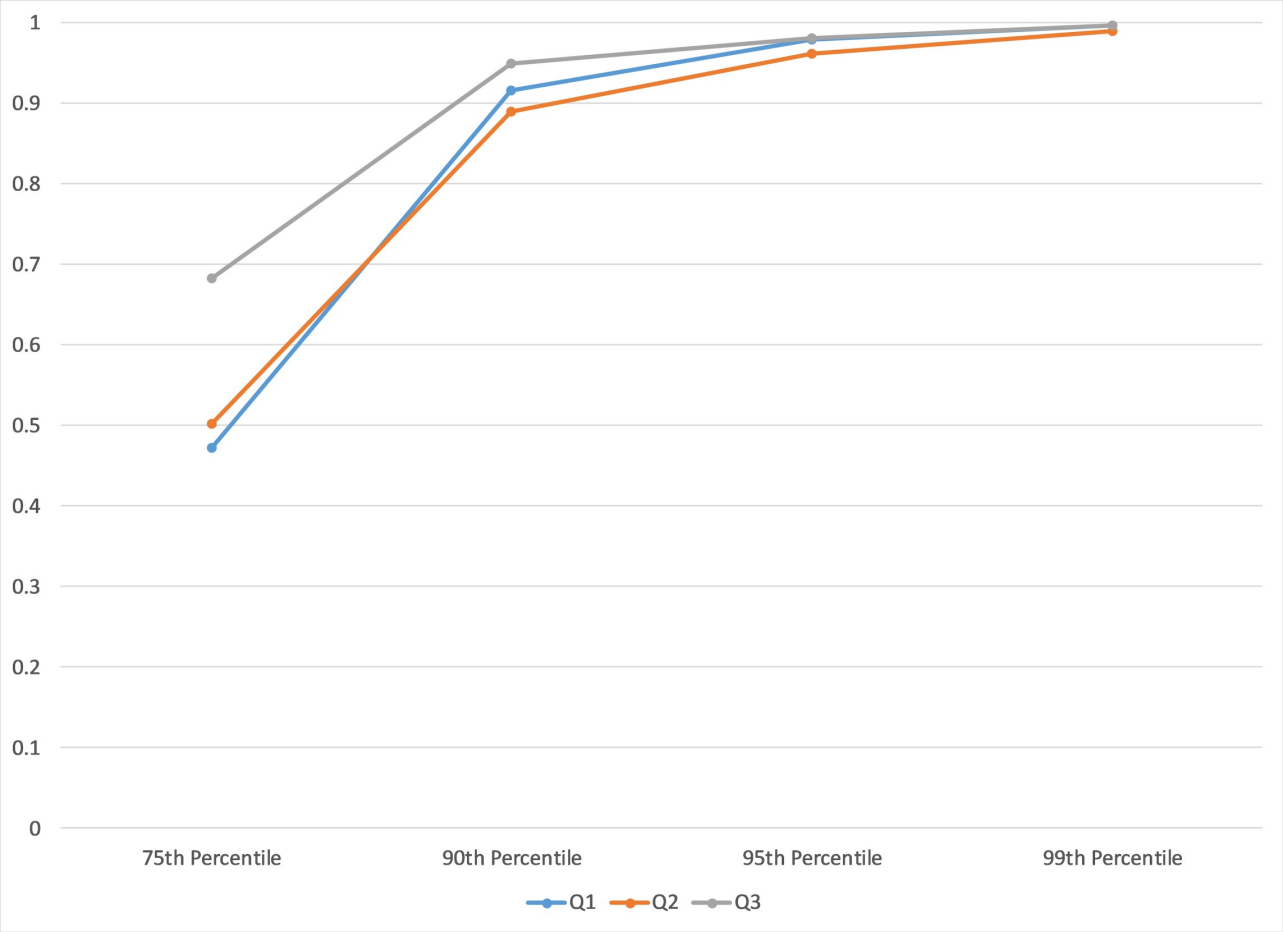
Probability establishment is a substantive innovator for 75^th^-99^th^ percentile by arts-design index bins (Q1-Q3).

Relevant probabilities from the 3 bin partition examining innovation orientation are graphed in Fig 4.

The 3-bin partition by ArtsDesignIndex to examine innovation orientation effectively differentiates those ZIP codes with the highest level from the two lower levels. The top-level ZIP codes, in terms of the ArtsDesignIndex, can be expected to have the top quarter or more of their establishments in tradable industries classified as substantive innovators while ZIP codes in the 2 lower levels only have even odds at the 75^th^ percentile. The suggestion that substantive innovators are reliant on strong web interest in design or arts avocations may or may not be true. The only thing we can conclude from this exercise is that the ArtsDesignIndex is unable to differentiate places where substantive innovators are relatively common from places where substantive innovators are very rare.

**Fig 5:**
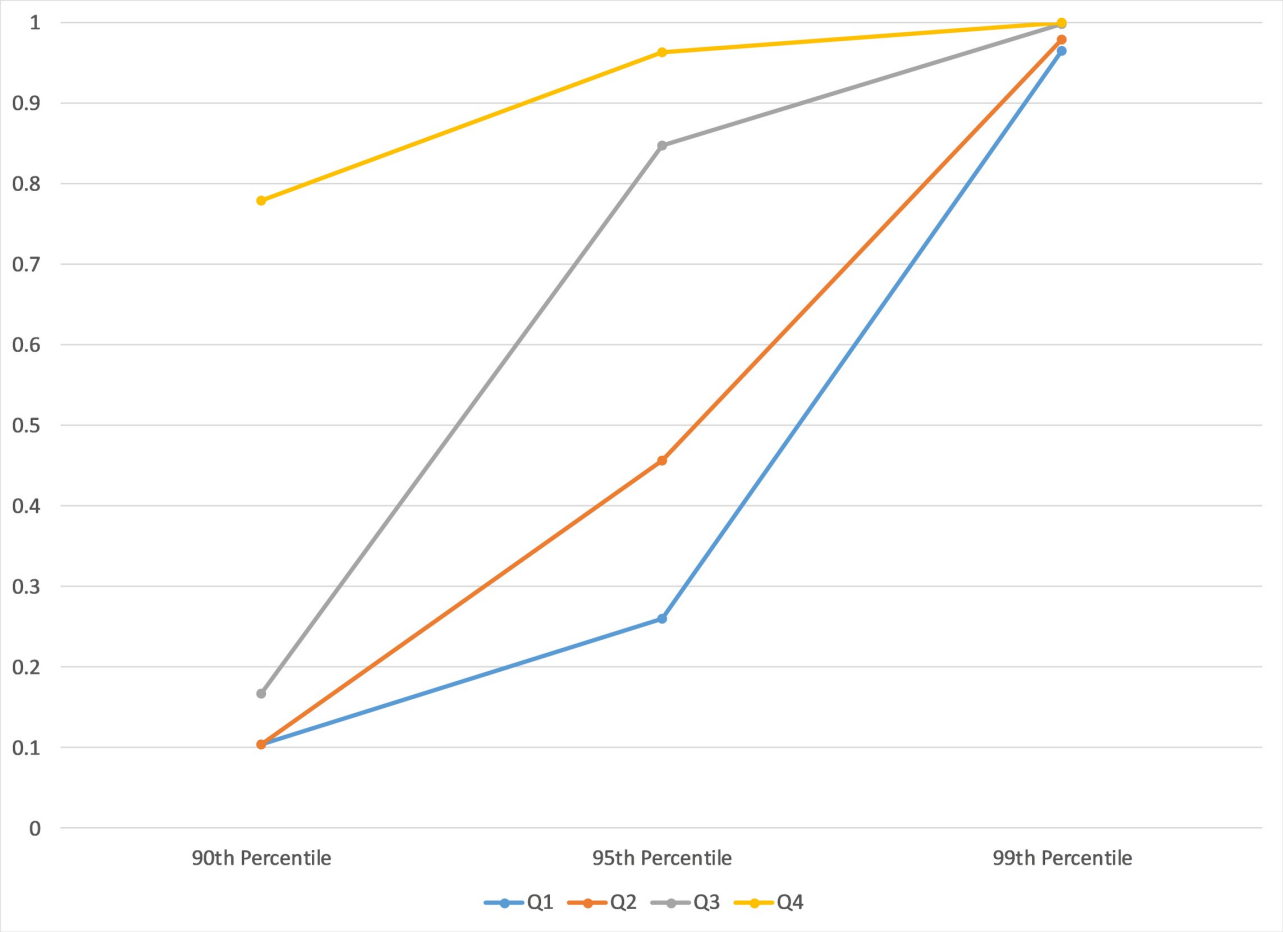
Probability establishment is design integrated for 90^th^-99^th^ percentile by arts-design index.

Relevant probabilities for the 4-bin partition examining design orientation are graphed in Fig 5.

In contrast, the differentiation across the 4 bins with respect to the design orientation of establishments does allow distinguishing where design integrated establishments are likely to be relatively common from places where design integrated establishments are likely to be very rare using the ArtsDesignIndex. The fact that substantive innovators are 3 to 4 times more numerous than design integrated establishments likely makes the differentiation problem easier in the latter case. Perhaps more importantly, one of the crafted audiences used to construct the index captures web interest in design problems. Given this result it might be possible to construct a crafted audience that explicitly captures web interest in innovation problems. Such an index, perhaps in combination with the ArtsAvocationIndex, might do a better job of identifying where substantive innovation is rare.

The bin exercise increases confidence that the ArtsDesignIndex is capturing something real with respect to design orientation because there appears to be a robust relationship with the probability an establishment is classified as design integrated. However, validation of the ability of the ArtsDesignIndex to identify where high-performance businesses may be much more common should come from outside the sample used to identify the relationship. We do this by assigning threshold values to the quantiles used in REIS to assign ZIP codes with an ArtsDesignIndex value not included to REIS to the appropriate bin. Bin membership for the full set of ZIP codes is then merged with the NETS dataset, allowing us to examine whether the index tends to be associated with a greater number of high growth establishments. The values for the bin thresholds and the expected share of design integrated and substantive innovators generated in REIS is provided in Table 6.

**Table 6:** Determining threshold values for ZIP codes using results from REIS analysis.

Quantile	High Growth Establishments Mean	High Growth Establishments 25th Percentile	High Growth Establishments Median	High Growth Establishments 75th Percentile
InnovLev1	3.417	0	1	4
Innov:ev2	4.42	0	1	5
InnovLev3	9.08	0	3	13
DesignLev1	3.38	0	1	4
DesignLev2	3.85	0	1	4
DesignLev3	5.3	0	1	6
DesignLev4	10.16	1	4	15

Source: 2014 ERS Rural Establishment Innovation Survey, DStillery Custom Crafted Audience.

If the expectation is that a larger number of substantive innovators or design integrated establishments would be more likely to spawn high growth establishments (“gazelles”) then NETS ZIP codes in the higher quantiles should have a larger number of these establishments. High growth establishments were identified using the definition of Clayton et al. [17], as employed by Choi et al. [18]. Clayton et al. [17] proposed a modified definition to the popular OECD definition of high growth. The OECD specified that high-growth establishments are those with 10 or more employees, and that experienced employment growth greater than 72.8% over a 3 year period [19]. However, this excludes small establishments, which may still experience high growth. Clayton et al. [17] proposed an alternative that incorporates both dimensions of relative and absolute growth by additionally classifying establishments with less than 10 employees as high growth if they experience an increase of 8 employees over a 3 year period. We follow this definition and classify establishments as high growth if they satisfy either criterion.

**Fig 6:**
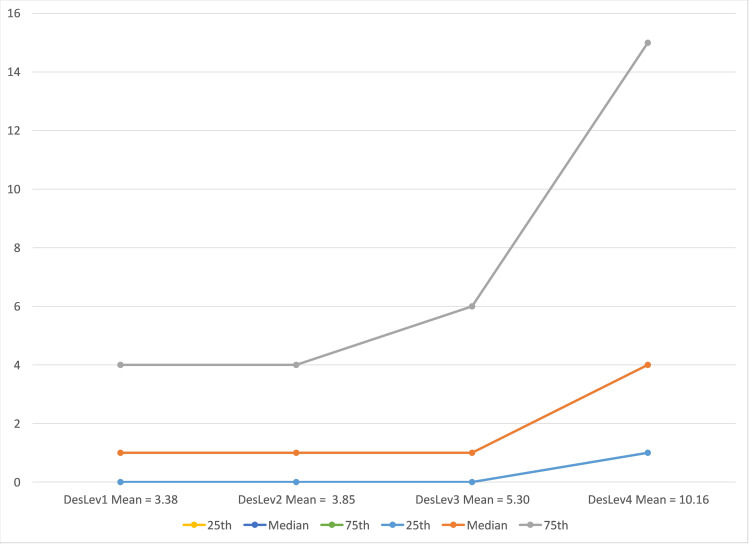
Number of high growth establishments in ZIP code.

The results for the 4-bin DesignLev categories are provided in Fig 6, including the median and interquartile range of the number of high growth establishments for each bin. Due to many ZIP codes with no high growth establishments, and a few ZIP codes with a very large number, the standard deviations around the means are too large to confirm a statistically significant difference between bins. However, given that the analysis is conducted on a census of establishments the lack of statistical significance is arguably moot. What the broad band of the high growth establishment distribution tells us is that nearly a quarter of all ZIP codes have no high growth establishments and more than a quarter of ZIP codes have 4 or more high growth establishments. However, more than half of the ZIP codes in the ArtsDesignIndex top quartile will have at least 4 high growth establishments, with the average number of such establishments being roughly twice that of the DesLev3 quartile and 3 times that of the DesLev1 quartile. The implications of these findings for framing regional development strategies are discussed in the concluding section.

## Using the entrepreneurship index to explain difference in start-up rates in NETS

The value of the REIS dataset for studying differences in start-up behavior is limited given the study population of interest (establishments with 5 or more employees) that makes a relatively rare event even rarer. Differences in start-up rates across space in the data are much more likely to be due to the luck of the draw in sample selection and completed responses than due to community characteristics that might facilitate new firm formation. The NETS data is much more appropriate for examining whether web interest in entrepreneurship topics in an area is associated with faster start-up rates. NETS covers all businesses with a formal credit relationship and does not impose any employment size limit. While the formal credit relationship criterion excludes a significant number of start-ups in any given year, an argument can be made that the coverage problem is more serious for low-quality start-ups. In addition, some of the “identified start-ups” may be already existing businesses that established a formal credit relationship for the first time in a given year. Despite these limitations, no other publicly available dataset provides a better view of potential start-up activity at the local level.

A serious empirical challenge to isolating an independent effect of the E-Ship Index on the start-up rate is the observed persistence in start-up rates across space [20]. Thus, while a simple regression (not shown) of the E-Ship Index on the start-up rate (number of start-ups divided by total number of establishments in ZIP code) produces a highly significant estimate of 1.7%, this only explains 3% of the variation in the dependent variable. While it is encouraging that the E-Ship Index performs as expected, the magnitude of the effect should be regarded with skepticism.

If start-up rates are persistent due to path dependency, then web behavior at any given time may be relatively inert in predicting future start-up rates. Alternatively, if start-up rates are persistent due to underlying explanatory variables that vary little over time then a seeming impact of web behavior on start-up activity may merely be capturing an association between current web activity and those underlying variables. Unfortunately, we do not have data at the ZIP code level of possible underlying factors to directly test this hypothesis. However, the data available at the ZIP code level will let us control for persistence in start-up rates to get a better handle on the unique contribution of web activity to entrepreneurial activity.

Results from a start-up rate regression including lagged start-up rates and indicators from the bottom two and top two quintiles of the E-ship Index are provided in Table 7. The dependent variable is expressed in percentage terms, so coefficient estimates below 1 represent a less than 1% change in the start-up rate. The magnitude of the highest quintile coefficient estimate is modest, suggesting that areas that demonstrated the highest level of entrepreneurship web activity could be expected to have start-up rates about a quarter of a percentage point higher than where this activity was moderate. Comparing the highest to the lowest quintile suggests a difference in roughly a half a percentage point. Given that the average start-up rate is about 1.6% this difference is not trivial. Another way to assess the magnitude is to compare the effect of web activity with the lagged start-up rate effect. The cumulative effect of lagged start-up rates is 0.3721, so at the mean level (1.6 x 0.3721 = 0.5234) the effect of lagged start-up rates is about twice as large an effect as moving from the middle to highest quintile in the E-ship index, and roughly a sixth larger than moving from the lowest to the highest quintile. Given that persistence in the start-up rate is recognized as an empirical regularity, the results suggest that local interest in entrepreneurship expressed as web activity may also be important for understanding differences in entrepreneurial activity across space.

**Table 7:** Regression of 2013 start-up rate on lagged start-up rates and E-ship index quintiles.

VARIABLES	Start-up Rate 2013
Start-up Rate 2009	0.0472***
	(0.00335)
Start-up Rate 2010	0.0609***
	(0.00145)
Start-up Rate 2011	0.123***
	(0.00393)
Start-up Rate 2012	0.141***
	(0.00423)
Lowest Quintile E-Ship Index	-0.189***
	(0.0201)
Second Quintile E-ship Index	-0.0585***
	(0.0201)
Fourth Quintile E-ship Index	0.0908***
	(0.0201)
Highest Quintile E-ship Index	0.260***
	(0.0205)
Constant	0.179***
	(0.0252)
Observations	27,552
R-squared	0.268

Standard errors in parentheses.

*** p<0.01, ** p<0.05, * p<0.1.

Source: National Establishment Time Series and DStillery.

## Constructing real time indicators of the entrepreneurial proclivities and the fervor of design and artistic imagination in a place

The strongest evidence that the E-ship, Design, and Arts Avocation indexes are picking up something real in local economies is the precision with which differences in start-up rates or design and innovation orientation are explained by them. Statistical significance is often accorded too much importance in assessing research findings, but that yardstick is particularly pertinent in assessing the value of indicators derived from nontraditional sources. And even more so when indicators are of things that conventional economic theorizing tend to dismiss out of hand such as the importance of design and the arts to innovation processes.

Of course, the reason why statistical significance is often irrelevant to the importance of a finding is because it tells us nothing about the importance of the statistical association, either in terms of magnitude or causality. The issue of magnitude is critically important to assess given large samples size in NETS, and to a lesser degree in REIS, where statistical significance may characterize phenomena that are economically trivial. Magnitude is a critical consideration for indicators to avoid delineating distinctions without real differences. This issue is addressed in more detail below. The issue of causality, however, is much less important in assessing the value of an indicator because there is no presumption that the variable of interest will be used as a lever to affect change.

In applying the DStillery produced indexes to the NETS data, the magnitude or salience of the associations is a valid concern. If the E-ship index is only associated with a quarter or half percent increase in start-up rates and the ArtsDesignIndex cannot differentiate between where high growth businesses will or will not be present, then how useful are they for local economic development decision-making? If predicting the outcomes from policy is the objective, then their value is questionable. The indexes are arguably much more valuable in identifying potentials and constraints that might be amplified or mitigated by policy. For example, there is a body of anecdotal evidence suggesting that design can be a powerful source of competitive advantage in rural as well as urban areas [21]. Pursuing an initiative like Handmade in America [22]—linking craftsmen, architects, and builders—might make sense in areas scoring high on the ArtsDesignIndex but would likely face an uphill battle in those places demonstrating little interest in design. Similarly, the E-ship index might provide insight on the most critical needs for promoting entrepreneurship. Is an area endowed with a large number of latent entrepreneurs in need of technical assistance to increase the launch rate? Or is entrepreneurship a relatively foreign construct, requiring stronger efforts in entrepreneurial education? Local economic development leaders on the ground may have some intuition regarding the local interest in entrepreneurship, design, or arts avocations but hard data derived from residents’ web behavior may provide a better basis for decision-making or for securing grants for economic development initiatives.

## Supporting information

S1 Appendix Representative websites suggested as starting points for identifying entrepreneurial propensities. (DOCX)

S2 Appendix Websites suggested as starting points for identifying arts avocation and design propensities. (DOCX)

## References

[1] Glaeser EL, Kim H, Luca M. Nowcasting the local economy: Using Yelp data to measure economic activity. NBER Working Paper 24010. Available from: https://www.nber.org/papers/w24010. 2017; 1–34.

[2] Kitchin R, Lauriault TP, McArdle G. Knowing and governing cities through urban indicators, city benchmarking and real-time dashboards. Regional Studies, Regional Science 2015; 2(1): 6–28. Available from: 10.1080/21681376.2014.983149

[3] Feldman M, Kogler D. Stylized facts in the geography of innovation In Hall BH, Rosenberg N, editors. Handbooks in the economics of innovation Volume 01 Amsterdam: Elsevier; 2010 pp. 381–410.

[4] Delgado M, Porter ME, Stern S. Clusters, convergence, and economic performance. Res. Policy 2014; 42(10):1785–1799.

[5] Packalen M, Bhattacharya J. Cities and ideas. NBER Working Paper No. 20921. 2015; 1–19. Available from: http://www.nber.org/papers/w20921 10/20/15.

[6] Dotzel KR, Faggian A. The impact of knowledge management strategies on innovation outcomes of rural and urban businesses in the United States. Growth Change 2019; 50(2): 515–547.

[7] Galindo-Rueda F, Millot V. Measuring design and its role in innovation. OECD Science, Technology and Industry Working Paper 2015/01. 2015; 1–51.

[8] Wojan TR, Nichols B. Design, innovation, and rural creative places: are the arts the cherry on top, or the secret sauce?” PLoS ONE 2018 13(2): e0192962 10.1371/journal.pone.0192962 29489884 PMC5831055

[9] Root-Bernstein R, Allen L, Beach L, Bhadula R, Fast J, Hosey C, et al Arts foster scientific success: avocations of Nobel, National Academy, Royal Society, and Sigma Xi members. J. of Psychology of Science and Technology 2008; 1(2): 51–63.

[10] LaMore R, Root-Berstein R, Root-Berstein M, Schweitzer JH, Lawton JL, Roraback E, et al Arts and crafts: critical to economic innovation. Econ. Dev. Q 2013; 27(3):221–229.

[11] Raeder T, Stitelman O, Dalessandro B, Perlich C, Provost F. Design principles of massive, robust prediction systems. Proceedings of the 18th ACM SIGKDD International Conference on Knowledge Discovery and Data Mining (KDD '12) 2012; 1357–1365. New York: Association for Computing Machinery. 10.1145/2339530.2339740

[12] Lenz P, Ibarra P. Using digital signals to measure audience brand engagement at major sports events: the 2015 MLB season. MIT Sloan Sports Analytics Conference, 2016 March, Boston. Available from: https://dstillery.com/wp-content/uploads/resources/white_paper/measure-audience-brand-engagement-at-major-sports-events.pdf

[13] Bottou L, Bousquet O. The tradeoffs of large-scale learning. Proceedings from Neural Information Processing Systems. 2007. Available from: http://papers.nips.cc/paper/3323-the-tradeoffs-of-large-scale-learning.pdf.

[14] Stitelman OM, Riesz C, Hook R, Dalessandro, B. Methods, systems and media for detecting non-intended traffic using co-visitation information. U.S. Patent No. 8,719,934. 2014.

[15] Florida R. The rise of the creative class: and how it’s transforming work, leisure, community, and everyday life. New York: Basic Books; 2002.

[16] Wojan T, Parker T. Innovation in the rural nonfarm economy: its effect on job and earnings growth, 2010–2014. Economic Research Report 237. Washington, DC: Economic Research Service; 2017.

[17] Clayton RL, Sadeghi A, Spletzer JR, Talan DM. High-employment-growth firms: defining and counting them. Monthly Lab. Rev. 2013 6; 136: 1–14.

[18] Choi T, Rupasingha A, Robertson JC, Leigh NG. The effects of high growth on new business survival. The Review of Regional Studies 2017; 47(1): 1–23.

[19] Statistical Office of the European Communities. Eurostat-OECD Manual on Business Demography Statistics. Paris: OECD Publishing 2007

[20] Andersson M, Koster S. Sources of persistence in regional start-up rates—evidence from Sweden. J of Economic Geography 2010; 11:179–201.

[21] Rosenfeld S. Manufacturing by design. Econ. Dev. Q. 2018, Vol. 32(4): 313–325

[22] McGehee NG, Knollenberg W, Komorowski A. The central role of leadership in rural tourism development: a theoretical framework and case studies. J Sustain Tourism 2015 23(8–9): 1277–1297.

