# Supplementary material for: Are the problem spaces of economic actors increasingly virtual? What geo-located web activity might tell us about economic dynamism
Source: PLoS One. 2020 Sep 17;15(9):e0239256. doi: 10.1371/journal.pone.0239256 (PMC7498009; doi:10.1371/journal.pone.0239256)
Supplement: S1 Appendix — (DOCX) [file pone.0239256.s001.docx]

## S1 Appendix: Representative Websites Suggested as Starting Points for Identifying Entrepreneurial Propensities

bplans.com

capterra.com

contentmarketinginstitute.com

ama.org

nrf.com

bridgespan.org

ssir.org

cmswire.com

mbaskool.com

managementhelp.org

ycombinator.com

martechtoday.com

smartpassiveincome.com

targetmarketingmag.com

openviewpartners.com

expandedramblings.com

socialmediatoday.com

digitalistmag.com

prsa.com
