# Supplementary material for: Are the problem spaces of economic actors increasingly virtual? What geo-located web activity might tell us about economic dynamism
Source: PLoS One. 2020 Sep 17;15(9):e0239256. doi: 10.1371/journal.pone.0239256 (PMC7498009; doi:10.1371/journal.pone.0239256)
Supplement: S2 Appendix — (DOCX) [file pone.0239256.s002.docx]

## Appendix 2: Websites Suggested as Starting Points for Identifying Arts Avocation and Design Propensities

### Arts Avocation Sites:

Music:

<http://imslp.org/>

<https://musopen.org/>

<https://www.8notes.com/>

<https://www.musicnotes.com/>

<https://musescore.com/>

<https://www.sheetmusicplus.com/>

<https://www.alfred.com/>

<https://irealb.com/>

<http://www.jazzbooks.com>

<https://www.learnjazzstandards.com/>

<https://www.jazzadvice.com/>

<https://www.freejazzlessons.com/>

<https://www.pianogroove.com/>

<http://www.jazz.org/>

<https://www.allaboutjazz.com/>

<http://downbeat.com/>

<https://www.allmusic.com/>

<https://www.discogs.com/>

<https://bandcamp.com/>

<https://www.shazam.com/>

<https://www.musixmatch.com/>

<http://www.jazz-sax.com/>

<http://www.ewimusician.com/>

<http://www.deepdishdesigns.com/>

<http://igigbook.com/>

<https://www.guitartricks.com/>

<https://www.guitarhabits.com/>

<https://www.uberchord.com/>

<http://www.guitarnick.com/>

<https://www.cyberfret.com/>

<https://nationalguitaracademy.com/>

<https://truefire.com/>

<http://artistworks.com/>

<http://www.traditionalmusic.co.uk/>

<http://www.chordie.com/>

<https://www.azchords.com/>

<https://www.songsterr.com>

<http://www.guitaretab.com/>

<http://www.violinist.com/>

<http://stringsmagazine.com/>

Top Ranked Online Musical Instrument Stores

<https://reverb.com/>

<https://www.sweetwater.com/> `

<http://www.guitarcenter.com/>

<http://www.musiciansfriend.com/>

Fine Art:

<https://www.artistsnetwork.com/>

<http://emptyeasel.com/>

<http://willkempartschool.com/>

<http://www.winsornewton.com/>

<http://www.wetcanvas.com/>

<https://www.dickblick.com/>

<https://www.cheapjoes.com/>

<https://www.utrechtart.com/>

<https://www.art-is-fun.com/>

<http://www.jerrysartarama.com/>

<https://www.art.com/>

Weaving, Crocheting, Quilting, needlepoint or Sewing

<https://www.paradisefibers.com/>

<https://woolery.com/>

<https://weavolution.com/>

<https://schachtspindle.com/>

<https://halcyonyarn.com/>

<https://www.yarn.com/>

<https://jimmybeanswool.com/>

<https://yarnsub.com/>

<https://www.loveknitting.com/>

<https://www.allfreeknitting.com/>

<http://www.yarnspirations.com/>

<http://thecrochetcrowd.com/>

<https://www.allfreecrochet.com/>

<http://www.redheart.com/>

<http://lionbrand.com/>

<https://needlepoint.com/>

Pottery, Ceramics or Jewelry

<https://ceramicartsnetwork.org/>

<http://www.theceramicshop.com/>

<http://clay-king.com/>

<https://www.bigceramicstore.com/>

<http://lakesidepottery.com/>

Leatherwork, metalwork or Woodwork

<http://www.fouroakscrafts.com/>

<http://www.instructables.com/>

<https://www.adafruit.com/>

<http://www.myhomefoundry.com/>

<http://backyardmetalcasting.com/>

<http://foundry101.com/>

<http://smallfoundrysupply.com/>

<https://centerforartinwood.org/>

<https://www.simplewoodturningtools.com/>

<http://monster-lathe-tools.com/>

<http://jtturningtools.com/>

<http://www.theokspindoctor.com/>

<http://derrytools.com/>

<http://thesandingglove.com/>

<https://www.tandyleather.com/>

<http://leatherworker.net>

<http://www.goldbarkleather.com/>

<http://howtoleather.com/>

<http://www.leathercraftlibrary.com/>

<http://sbearstradingpost.com/>

Photography as an Art

<https://www.flickr.com/>

<https://digital-photography-school.com/>

<https://photographylife.com/>

<http://kenrockwell.com/>

<https://petapixel.com/>

<https://fstoppers.com/>

<https://www.slrlounge.com/>

<https://improvephotography.com/>

Dance:

<http://www.learntodance.com/>

<https://www.passion4dancing.com/>

<https://www.danceplug.com/>

<http://www.dancetothis.com/>

<https://www.danceclass.com/>

<http://www.balletforadults.com/>

<https://dancer.com/>

<https://www.russianpointe.com/>

<http://www.adult-ballet.org/>

<http://adultballerinaproject.com/>

<http://onlineballetclass.com/>

<https://www.idance.net/>

<http://hiphopcrusher.com/>

<https://www.brambilabong.com/>

<https://www.steezy.co/>

<http://www.danceadvantage.net/>

<http://www.dancespirit.com/>

<http://www.dance.net/>

Acting and Theatre”

<https://www.backstage.com/>

<http://www.projectcasting.com/>

<http://www.auditionsfree.com/>

<http://www.2018auditions.com/>

<http://www.playbill.com/>

<https://www.broadwayworld.com/>

<https://takelessons.com/>

<http://www.communitytheater.org/>

<http://produceaplay.com/>

<http://studio-productions-inc.com/>

<http://smnetwork.org/>

<http://actorsequity.org/>

<http://www.sagaftra.org/>

<http://actorsaccess.com/>

<https://home.castingnetworks.com/>

<http://lort.org/>

<https://theatrenerds.com/>

<https://performerstuff.com/>

<http://www.ace-your-audition.com/>

<http://www.actorhub.co.uk/>

<https://musicaltheatreresources.com/>

Creative Writing:

<https://www.writingforward.com/>

<http://thinkwritten.com/>

<https://thejohnfox.com/>

<https://www.plot-generator.org.uk/>

<https://letswriteashortstory.com/>

<http://www.bryndonovan.com/>

<http://writingexercises.co.uk>

<http://springhole.net/>

<http://www.seventhsanctum.com>

<http://chaoticshiny.com/>

<http://www.namegeneratorfun.com/>

<http://www.aspenwords.org/>

<https://ox.libguides.com/>

<https://www.poetrysoup.com/>

<https://www.poemhunter.com/>

<https://allpoetry.com/>

<http://poetry.com/>

<https://deepundergroundpoetry.com/>

<http://www.digitalpoet.net/>

<https://poetryslam.com/>

Media Arts:

<https://www.filmsourcing.com/>

<http://howtofilmschool.com/>

<http://www.theblackandblue.com/>

<http://www.filmtools.com/>

<http://www.cinematography.com/>

<http://www.thehurlblog.com/>

<http://noamkroll.com/>

<http://www.dvxuser.com/>

<http://www.indiewire.com/>

Opera:

<http://www.theoperadatabase.com/>

<https://www.operaamerica.org>

<http://aria-database.com/>

### Design Sites:

<https://befonts.com/>

<https://freedesignresources.net/>

<https://www.canva.com/>

<https://www.befunky.com/>

<https://www.picmonkey.com/>

<https://www298.lunapic.com/>

<http://www.flatuicolorpicker.com/>

<https://www.designspiration.net/>

<http://abduzeedo.com/>

<https://www.siteinspire.com/>

<https://twibfy.com/>

<https://www.ideo.com/>

<https://www.ideo.org/>

<https://www.ideou.com/>

<http://designtaxi.com/>

<https://muz.li/>

<https://uxdesign.cc/>

<http://www.prototypr.io/>

<https://uxplanet.org/>

<https://niice.co/>

<http://creatively-daring.com/>
